# Evolutionary fates of gene‐body methylation and its divergent association with gene expression in pigeonpea

**DOI:** 10.1002/tpg2.20207

**Published:** 2022-07-05

**Authors:** Alim Junaid, Nagendra Kumar Singh, Kishor Gaikwad

**Affiliations:** ^1^ National Institute of Plant Biotechnology Pusa Campus New Delhi 110012 India

## Abstract

Pigeonpea (*Cajanus cajan* L. Huth) is an agronomically important legume cultivated worldwide. In this study, we extensively analyzed gene‐body methylation (GbM) patterns in pigeonpea. We found a bimodal distribution of CG and CHG methylation patterns. GbM features‐ slow evolution rate and increased length remained conserved. Genes with moderate CG body methylation showed highest expression where as highly‐methylated genes showed lowest expression. Transposable element (TE)‐related genes were methylated in multiple contexts and hence classified as C‐methylated genes. A low expression among C‐methylated genes was associated with transposons insertion in gene‐body and upstream regulatory regions. The CG methylation patterns were found to be conserved in orthologs compared with non‐CG methylation. By comparing methylation patterns between differentially methylated regions (DMRs) of the three genotypes, we found that variably methylated marks are less likely to target evolutionary conserved sequences. Finally, our analysis showed enrichment of nitrogen‐related genes in GbM orthologs of legumes, which could be promising candidates for generating epialleles for crop improvement.

## Core Ideas

- We classify, for the first time, the genes of pigeonpea based on their evolutionary origins.
- We characterized the influence of methylation on the mode of origins in pigeonpea.
- Differentially methylated regions are less likely targets of evolutionary conserved sequences in pigeonpea.
- Gene‐body methylation exerts a heterogeneous effect on gene expression.

**ABBREVIATIONS**
DMR: differentially methylated region
GbM: gene‐body methylation
GO: gene ontology
Ka: nonsynonymous substitution rate
Ks: synonymous substitution rate
LTR: long terminal repeat
RNA‐seq: RNA sequencing
SGD: single‐gene duplicate
TE: transposable element
WGD: whole‐genome duplication

## 1 INTRODUCTION

DNA methylation is an evolutionary conserved epigenetic mechanism in plants and animals that involves methylation of cytosine bases at position five of the cytosine ring and regulates gene expression and genome stability (Robertson, 2005; Slotkin & Martienssen, 2007). In contrast to animals, DNA methylation in plants (Wu & Zhang, 2010; Zhang & Zhu, 2012; Zhu, 2009) occurs in all cytosine contexts, that is, CG, CHG, and CHH (where H denotes A, T, or C) (Henderson & Jacobsen, 2007). The establishment and maintenance of context‐specific DNA methylation is regulated by different pathways in plants. DNA METHYLTRANSFERASE 1 (MET1), an ortholog of DNA (cytosine‐5‐)‐METHYLTRANSFERASE 1 (DNMT1) in mammals (Bestor et al., 1988), regulates methylation in CG context. A plant‐specific DNA METHYLTRANSFERASE CHROMOMETHYLASE 3 (CMT3) regulates methylation in CHG context. The CHH methylation involves a 24‐nt small RNA‐guided DNA methylation (RdDM) pathway and DOMAINS REARRANGED METHYLTRANSFERASE (DRM2) (Cao & Jacobsen, 2002; Chan et al., 2005). Different features of the genome are subjected to DNA methylation based on their function. Regulation of gene expression involves occurrence of DNA methylation at different positions within a gene, promoter, or the transcribed gene‐body. Methylation in promoter regions is profoundly associated with transcriptional repression (Domcke et al., 2015; X. Zhang et al., 2006; Zhu et al., 2016), but they have been shown to activate transcription in certain instances (Lang et al., 2017; Liu et al., 2015). Gene‐body methylation (GbM) occurs primarily in CG context within the coding regions of genes and is excluded from the transcriptional start and termination sites (Takuno & Gaut, 2013). Although the precise molecular mechanisms of GbM establishment remains opaque, its maintenance is proposed to depend on a CHG methyltransferase, which belongs to the *CHROMOMETHYLASE* gene family (Bewick & Schmitz, 2017). GbM has been proposed to play a role in enhancing the efficiency of pre‐messenger RNA splicing and regulate gene homeostasis by preventing transcription from cryptic promoters (Regulski et al., 2013; Takuno & Gaut, 2012; Zilberman et al., 2008). Notably, GbM has been also proposed to have evolutionary consequences. A comparative analysis of GbM between rice (*Oryza sativa* L.) and *Brachypodium distachyon* members of Poaceae family, which diverged at least 40–53 million years ago (MYA), revealed a conservation of GbM between orthologs that was further proposed to have a functional implication of targeting a subset of genes for methylation (Takuno & Gaut, 2013). A similar trend was also shown to occur between *Arabidopsis lyrata* and *A. thaliana* that diverged ∼26 MYA. GbM has been shown to target long and slowly evolving housekeeping genes, and this feature is conserved in angiosperms with an exception in Poaceae family where GbM genes have been shown to evolve at a faster rate (Niederhuth et al., 2016; Takuno & Gaut, 2012, 2013; X. Zhang et al., 2006). Furthermore, GbM is also proposed to impact evolutionary properties of genes in grasses and other monocots, such as their nucleotide landscape, particularly bimodal CG distribution (Carels & Bernardi, 2000; Serres‐Giardi et al., 2012; Takuno & Gaut, 2013).

Polyploidization is an important characteristic phenomenon of angiosperm evolution (Soltis et al., 2015). Most eudicots have been subjected to at least one polyploidization event (Akoz & Nordborg, 2019; The French–Italian Public Consortium for Grapevine Genome Characterization, 2007). Polyploidization has been proposed to condition important genetic traits for domestication for agricultural improvement (Salman‐Minkov et al., 2016). In addition, plant genomes are also prone to more local or segmental duplication events that helps to generate new or expand existing gene sets (Cannon et al., 2004). Since genomes of angiosperms are prone to polyploidization arising from whole‐genome duplication (WGD) events, they have also evolved a tendency for diploidization (to prevent predictive extinction) and for duplicated gene loss over long evolutionary periods (Coghlan et al., 2005; Otto & Whitton, 2000; Paterson et al., 2010; Wolfe, 2001). Duplicated genes may face two evolutionary fates: (a) biased retention of both copies owing to neo‐functionalization by accumulating neutral mutations or gene balance via fractionation to single copy (Lewis 1951, Ohno 1970) and (b) selective loss of one of the paralogous genes. However, the knowledge on the influence of the epigenome on these evolutionary events is limited. Recent advances in genome and epigenome sequencing have shed light on WGD events in different plants and their epigenetic landscape. For example, it has been found that a WGD event occurred in soybean [*Glycine max* (L.) Merr.] ∼13 MYA with no immediate diploidization. Because of this duplication, 75% of soybean genes are present in multiple copies (Schmutz et al., 2010). These duplicated genes are enriched in CG GbM and show relatively higher expression levels than singleton genes (Kim et al., 2015, 2009; Schmutz et al., 2010). Thus, although there is developing evidence of the influence of methylation on evolutionary fates of duplicated genes; however, such knowledge is restricted to only a few selective crops that are widely used for human consumption.

**Core Ideas**

- We classify, for the first time, the genes of pigeonpea based on their evolutionary origins.
- We characterized the influence of methylation on the mode of origins in pigeonpea.
- Differentially methylated regions are less likely targets of evolutionary conserved sequences in pigeonpea.
- Gene‐body methylation exerts a heterogeneous effect on gene expression.

Pigeonpea (*Cajanus cajan* L. Huth) is an agronomically important crop that serves as protein source for human consumption belonging to subfamily Papilionoidea of the legume family. It is widely cultivated in ∼4.23 million ha. worldwide (FAO), thus also providing a source of livelihood to many farmers. Pigeonpea is a diploid species (2*n* = 22) with a genome size of ∼833 Mb. Its genome was also subjected to WGD events ∼58 MYA along with the other members of Papilionoidea (Varshney et al., 2011). But there is no record of a recent WGD event in pigeonpea unlike its close relative soybean. However, there is evidence of more local rearrangements occurring in pigeonpea during evolution (Varshney et al., 2011). This makes pigeonpea a relatively simple model to investigate the potential relationship between methylation and evolution of duplicate genes.

In this study, we provide insights into the floral‐bud‐specific methylome of two naturally varying pigeonpea lines: 11A sterile, 303R fertile, as well as their F_1_ hybrid at a single‐base‐pair resolution. We specifically investigated roles of CG GbM conservation and divergence by exclusive analysis of CG GbM patterns. We also compared GbM within pigeonpea paralogs as well as orthologous genes between pigeonpea and its close relative soybean with a more recent WGD event. Lastly, we investigated the natural epigenetic divergence by comparing methylation patterns of variably methylated regions within the floral buds of the three pigeonpea genotypes and their evolutionary fate.

## 2 METHODS

### 2.1 Plant materials and growth conditions

Three different genotypes of pigeonpea, namely 11A (male sterile), 303R (fertile), and 11A × 303R (hybrid), were used for experiments and analysis (Junaid et al., 2018). Plants were grown in a greenhouse under natural conditions at the Indian Agricultural Research Institute New Delhi, India. Genotypes were verified by pollen fertility test using 2% acetocarmine dye as described previously (Junaid et al., 2018).

### 2.2 DNA isolation and bisulfite sequencing library preparation

Immature young buds were harvested from 3‐month‐old plants. Two biological replicates were used for each of the three lines. High molecular weight DNA was extracted using CTAB method with slight modifications (Porebski et al., 1997). The pair‐end bisulfite sequencing libraries were prepared using Illumina Truseq DNA library preparation kit according to manufacturer's instructions. Briefly, 1–5 μg of DNA was sheared to 100–300 bp using Covaris‐M220 and ensued DNA fragments were end repaired by adding dA at the 3′ end prior to the ligation of Truseq methylated adapters. The adapter ligated DNA fragments were treated with bisulfite using EZ DNA Methylation‐Gold kit (D5005) and sequenced using Illumina Hi‐Seq 2000 platform following manufacture's instruction.

### 2.3 Bisulfite sequencing data analysis

Raw reads containing adapters and low‐quality bases at the 5′ and 3′ end were cleaned using Cutadapt (Martin, 2011) and Trim galore (https://github.com/FelixKrueger/TrimGalore). High quality reads were mapped to the reference genome of pigeonpea (Varshney et al., 2011) and soybean (Schmutz et al., 2010) using BSMAP at default parameter (Xi & Li, 2009). The methylation status of each of the mapped cytosine bases was extracted using methratio.py (Xi & Li, 2009) and weighted methylation level was calculated. The metaplots analysis of genes and transposons were performed by dividing 2‐kb flanking and gene‐body region into equal size bin. Methylation levels were then calculated for each bin and plotted in ggplot2. Previously published data (SRR5850382, SRR1628892) of soybean methylome was used for analysis (Li et al., 2017).

### 2.4 Gene‐body methylation determination

The GbM of each gene was determined according to Takuno & Gaut (2012) with a modified strategy. Genes were classified into transposable element (TE) and non‐TE categories based on transposons insertion. The CG, CHG, and CHH methylation level in CDS regions were then calculated for both gene categories separately. We used a minimum coverage cutoff of 3× for CG and CHG and 10× for CHH context to calculate body methylation for genes. Afterwards, binomial probability distribution was applied to calculate one‐tailed *p* value based on divergence of CG methylation levels of a gene from average genomic methylation levels.

#### 2.4.1 Identification of differentially methylated regions

To identify differentially methylated region (DMRs) between three genotypes, methylkit (Akalin et al., 2012) pipeline was used. In brief, genome was tiled into 100‐bp windows, weighted methylation level was calculated for each window, and a pair‐wise test was performed. The filtering criteria was also adopted to exclude ambiguous cytosine resulting from incomplete conversion or sequencing error. We used cytosine with minimum 10× coverage and at least three cytosine for CG and CHG and six cytosines for CHH to define 100‐bp window. We used logistic regression model to calculate *p* values, which was further adjusted to false discovery rate using sliding linear model method for final DMR identification. The windows with methylation difference with at least 20% and *q* value <0.01 for all the three contexts were considered as candidate DMRs.

### 2.5 RNA‐seq library preparation

Total RNA from the bud of pigeonpea hybrid line was extracted using Sigma RNA extraction kit (E4913) according to the manufacturer's instructions. The RNA sequencing (RNA‐seq) libraries were prepared in two biological replicates using the standard TruSeq RNA sample preparation kit (Illumina) and sequenced in 100‐bp pair end on Illumina Hi Seq 2000 platform. For 11A and 303R lines of pigeonpea, we used publicly available data of 303R (SRR6780151, SRR6780152) and 11A (SRR6780153, SRR6780154) (Saxena et al., 2020).

### 2.6 RNA‐seq data analysis

Raw reads were filtered for adapter removal, the high‐quality reads were aligned to soybean and pigeonpea reference genomes using tophat2 (Kim et al., 2013) at default parameters. The ensued aligned reads were provided for transcript assembly and quantification of transcript abundance was performed using cufflinks. The transcript expression was determined in terms of fragments per kilobase of exon per million fragments mapped (fpkm). Transcripts were annotated against nonredundant NCBI data base using Blast2GO (https://www.blast2go.com). The gene ontology (GO) enrichment analysis was performed using AgriGO (http://bioinfo.cau.edu.cn/agriGO/), and enriched terms were defined with Fisher's exact test and false discovery rate correction by Bonferroni method (Du et al., 2010).

#### 2.6.1 Transposons identification

We used a combinational approach to identify robust sets of transposons in pigeonpea and soybean. The soybean transposons library was downloaded from SoyBase (Grant et al., 2010), while the repeat modeler was used to construct a de novo repeat library of pigeonpea genome. Afterwards, RepeatMasker (http://www.repeatmasker.org) was run at default parameters using repeat libraries to identify transposons. Transposons <100 bp were discarded from subsequent analysis. In addition to this, LTRdigest (Steinbiss et al., 2009) and LTRharvest (Ellinghaus et al., 2008) were also used to identify full‐length long terminal repeats (LTRs). The LTR data sets generated from both approaches were further compared and overlapped LTRs were considered for further analysis.

#### 2.6.2 Identification of homologous genes

Duplicated and syntenic gene pairs between pigeonpea and soybean were identified by using MCScanX (Wang et al., 2012). The proteome sequences of both species were downloaded from NCBI and reciprocal Blastp approach was adopted with parameter of *e* value 1 × 10^−10^ and output was generated in ‐m 8 format. The resulting tab‐delimited output was provided to MCScanX, and subsequently collinear regions were identified. The genes were classified into different duplicated categories using command duplicate_gene_classifier. Protein sequences of homologous genes were aligned and resulting alignment output was converted into axt format using clustaw2 (Thompson et al., 1994) and paraAT (Zhang et al., 2012). Then, nonsynonymous (Ka) and synonymous (Ks) substitution rate values were calculated by employing the method of Nei and Gojobori (1986) using KaKs_Calculator (Z. Zhang et al., 2006).

## 3 RESULTS

### 3.1 Genome‐wide landscape of DNA methylation in pigeonpea floral buds

**FIGURE 1:**
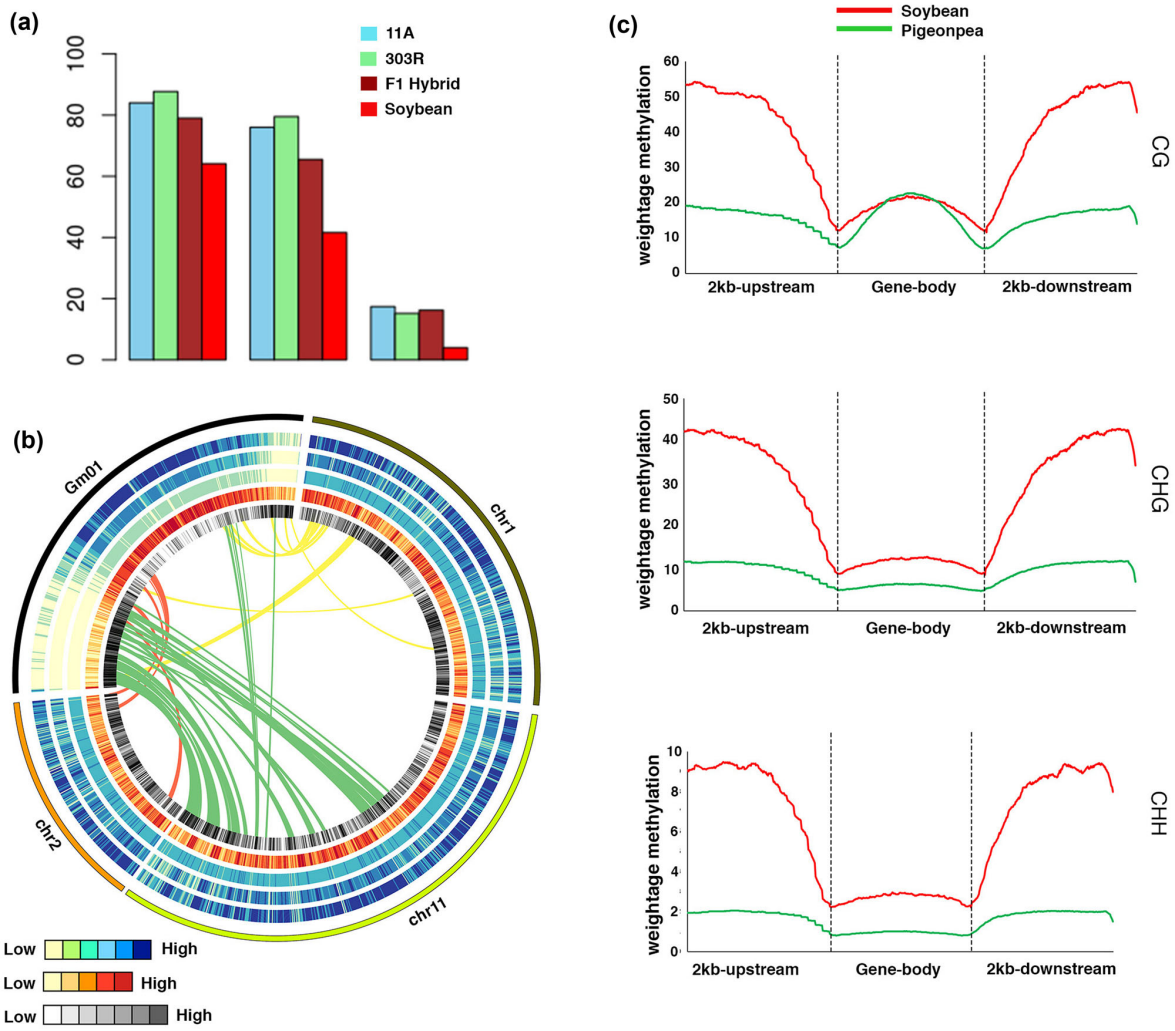
Comparative analysis of whole‐genome DNA methylation patterns of pigeonpea and soybean. (a) Comparison of genome‐wide DNA weighted methylation levels of flower buds in three different genotypes (11A, 303R, and F_1_ hybrid) of pigeonpea and soybean. (b) A circos plot showing the DNA methylation landscape and syntenic association of homologous gene pairs in soybean (Gm01) and pigeonpea (Chr1, Chr2, and Chr11). Heatmap shows CG, CHG, and CHH methylation levels, transposons density and gene density (outer to inner circle). Red and black colors indicate high transposons and gene densities, respectively. Blue color indicates high methylation levels and yellow color indicates low methylation level. (c) Metaplots showing CG, CHG, and CHH methylation pattern in gene‐body and flanking regulatory regions in pigeonpea and soybean (+2 kb upstream from transcriptional start sites and −2 kb downstream from transcriptional termination sites). Cc, *Cajanus cajan* (pigeonpea); Gm, *Glycine max* (soybean). The numbers indicate the respective chromosome numbers

We characterized and compared methylomes of floral buds of pigeonpea lines 11A (male sterile), 303R (fertile), and their F_1_ hybrid using two biological replicates at single‐base‐pair resolution using whole‐genome bisulfite sequencing. In total, 330 million reads were generated for all the three lines. After trimming adaptors and low‐quality reads, 72–84% cleaned reads were mapped to the pigeonpea reference genome (Varshney et al., 2011) (Supplemental Table S1). The bisulfite conversion efficiency was calculated by aligning reads to the unmethylated chloroplast and lambda genome. Thereafter, methylation levels in CG, CHG, and CHH contexts between biological replicates were compared in 2‐kb windows to check the variability within six sequenced libraries. A minimum coverage for cytosines in symmetric sites were considered to be at least four, whereas for asymmetric site it was increased to 10. We found high correlation in symmetric sites (*r* = 0.91–0.94, CG; *r* = 0.92–0.94, CHG) and relatively lower correlation in asymmetric sites (*r* = 0.71–0.86, CHH) (Supplemental Figure S1). We also observed a bimodal distribution of methylation at CG and CHG sites (Supplemental Figure S1). However, methylation levels in the majority of CHH sites were found to be highly reduced. A comparison of global DNA methylation patterns between three lines of pigeonpea revealed overall high CG and CHG weighted methylation levels in 303R line relative to 11A and F_1_ hybrid but higher CHH methylation in 11A followed by F_1_ and 303R. The weighted methylation levels varied from 78 to 84% in CG, 63 to 79% in CHG, and 14 to 17% in CHH context in 11A, 303R, and hybrid lines. These levels were much higher than those observed in floral buds of soybean (CG = 64.1%, CHG = 41.3%, and CHH = 4%) (Figure 1a), a close relative of pigeonpea that diverged ∼20 to 30 MYA. Genome‐wide landscapes of CG and CHG methylation showed a positive correlation with the transposon density (*r* = 0.23; CG, *r* = 0.24; CHG *p* value < 2.2 × 10^−16^) and a negative correlation with the gene density (*r* = −0.56; CG, *r* = −0.58; CHG *p* value < 2.2 × 10^−16^) in pigeonpea (Figure 1b). Chromosome‐wide distribution analysis of CHH methylation surprisingly revealed an unexpected methylation pattern in pigeonpea. Transposons‐rich regions showed a weak, although significant, negative association (*r* = −0.07; *p* value = 1.488 × 10^−11^), and gene‐dense regions showed a reduced positive association (*r* = 0.02; *p* value = 0.04) with CHH methylation levels. These patterns contrast those observed in soybean, where transposons‐rich centromeric regions showed relatively higher methylation levels than the gene‐dense telomeric regions of the chromosomes in all three cytosines (CG, CHG and CHH) contexts.

Genome‐wide DNA methylation analysis showed a substantially high methylation in pigeonpea compared with soybean. Therefore, we further analyzed the CG, CHG, and CHH weighted methylation levels of protein‐coding genes and TE regions in pigeonpea and soybean. Genes intersecting with transposons in gene‐body regions were removed from the subsequent metaplot analysis. The metaplots showed maximum distribution of CG, CHG and CHH methylation levels in the gene flanking regions, followed by a continuous drop with minima around transcriptional start sites and transcriptional termination sites (Figure 1c). Methylation levels increased continuously when moving away from these sites. We also observed a CG methylation peak within the gene‐body regions of both species, although it was relatively more prominent in soybean than pigeonpea. In contrast, CHG and CHH metaplots were marked by absence of this peak and methylation levels were uniform throughout the gene‐body region. Consistent with whole genome methylation levels, pigeonpea exhibited a higher methylation level in all three methylated cytosines contexts in flanking and gene‐body regions relative to soybean (Figure 1c).

Analysis of TE methylation patterns showed highest CG methylation levels in all TE categories for both pigeonpea and soybean. The methylation levels of CG within the TE bodies of DNA elements and retrotransposons (Copia, Gypsy) were equivalent in both species (Supplemental Figure S2A,D,J), indicating a robust maintenance of CG methylation in the TE body during evolution. Pigeonpea showed relatively higher CHG methylation levels in the TE body compared with soybean (Supplemental Figure S2B,E,H,K). Methylation levels in CG and CHG contexts in the TE‐body‐flanking regions of DNA elements, Gypsy and Copia, were also comparable between the two species. However, both CG and CHG methylation levels in the flanking regions of LINES transposons were relatively higher in pigeonpea (Supplemental Figure S2G,H). Notably, pigeonpea showed significantly high levels (4×) of CHH methylation in TE body as well as its flanking regions for all the TE categories (Supplemental Figure S2C,F,I,L).

### 3.2 Characterization of GbM patterns and associated gene features

**FIGURE 2:**
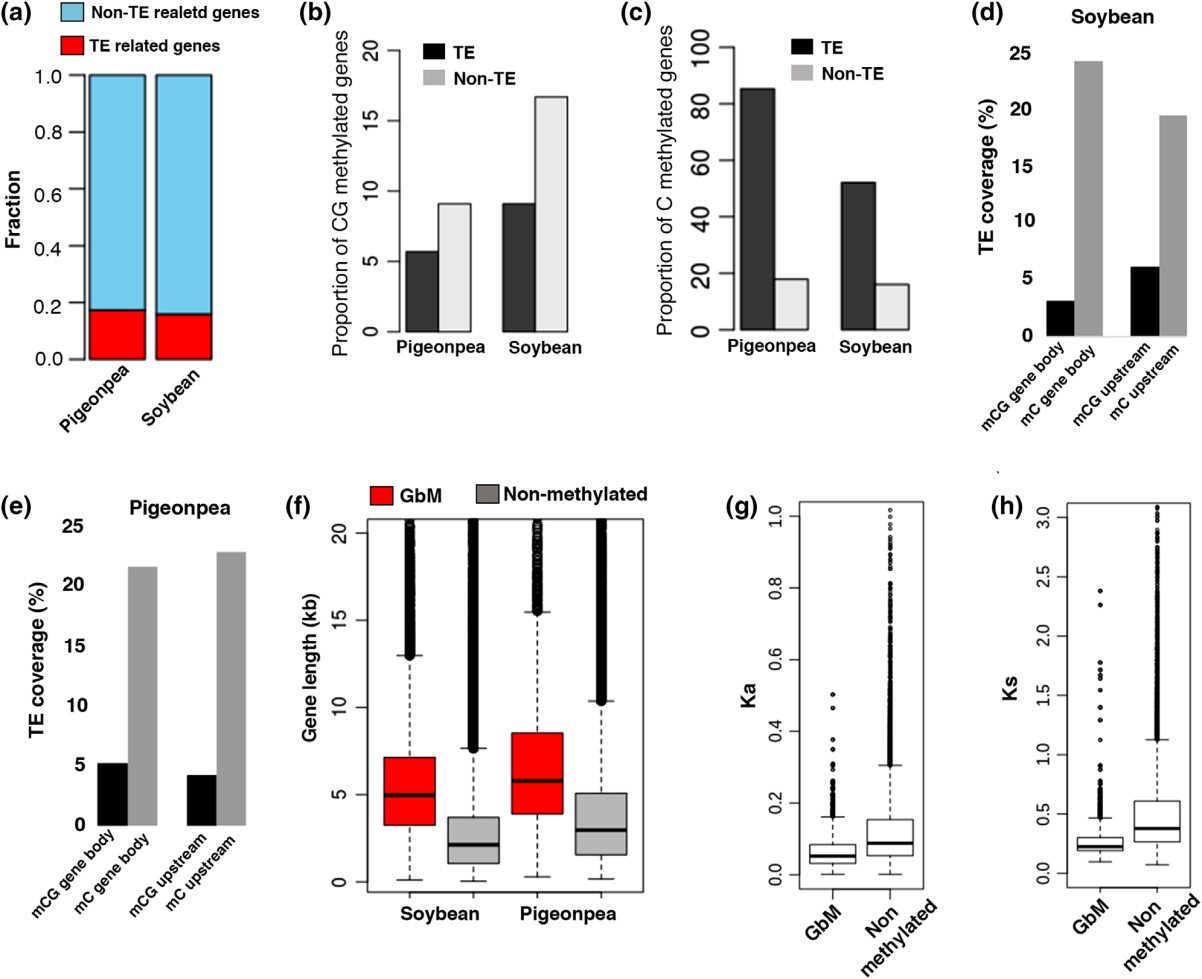
Characterization of distinct features of CG body and non‐CG methylated genes. (a) Classification of pigeonpea and soybean genes based on transposon insertion within gene‐body region. (b–c) Fraction of (b) mCG (c) non‐CG body methylated in the context of transposons insertion. The significance in distribution of CG body methylated genes was tested using Chi square test; *p* value < 2.2 × 10^−16^. (d–e). Percentage of transposons coverage in 2 kb upstream and body region showing high abundance of transposons in non‐CG methylated genes in (d) soybean and (e) pigeonpea. (f) Box plots showing gene length in gene‐body methylation (GbM) genes and non‐methylated genes in pigeonpea and soybean. Note higher length of GbM genes in both species relative to non‐methylated genes. (g–h) (g) Nonynonymous substitution (Ka) and (h) synonymous substitution rate (Ks) distribution of mCG body methylated and non‐methylated ortholog pairs. The CG body methylated genes showed low divergence relative to unmethylated genes. TE, transposable element

We next conducted an intricate analysis of genic methylation patterns in pigeonpea and also compared them with soybean. For this, genes were categorized based on the presence or absence of transposons (TE genes and non‐TE genes, respectively, hereafter). We found a much lower proportion of TE genes (17.3 and 15.8%) than non‐TE genes (82.6 and 84.1%) in both pigeonpea and soybean (Figure 2a). Thereafter, weighted methylation levels were calculated for both gene categories. In line with predictions that transposons insertion contributes to increased methylation levels, we found high levels of methylation within the gene body of TE genes relative to non‐TE genes in all mC (CG, CHG and CHH) contexts in both species (Supplemental Figure S3).

Next, in order to investigate the potential role of CG body methylation and their conservation during evolution, TE genes and non‐TE genes were separately classified into CG body methylated (GbM) and C‐methylated genes based on their genic background methylation level using a probabilistic approach (Takuno & Gaut, 2012). Genes that were significantly enriched in CHG and CHH methylation with *P*_CHG_ and *P*_CHH_ < 0.05 were categorized as C‐methylated genes. The GbM genes were identified with *P*_CG_ < 0.05 and methylated exclusively in CG context. We identified a total of 2,608 (8.5%); 2,343 (8.8%); 2,763 (10%); and 8,466 (15.5%) body‐methylated genes in 11A, 303R, F_1_ hybrid, and soybean, respectively. The distribution of GbM genes in TE and non‐TE genes was found to be significantly different in both species (*p* value < 2.2 × 10^−16^; Chi square test). Among GbM genes, a significant proportion in pigeonpea (2,245; 9.7% of total) and soybean (7,673; 16.7% of total) belonged to non‐TE genes, while only a smaller fraction of TE genes (5.7% in pigeonpea and 9.1% in soybean of total) were body methylated in both species (Figure 2b). We also observed that TE gene comprehended a high number of C‐methylated genes, wherein 56–84% of the genes were significantly methylated at multiple contexts (Figure 2c). Consistent with this, we found high TE coverage in gene‐body and upstream (2 kb) regions of C‐methylated genes in pigeonpea and soybean (Figure 2d,e).

Earlier studies in *Arabidopsis* and a few species of Poaceae family have shown a positive correlation between gene length or number of exons and CG body methylation, where significantly body methylated genes were longer than the lowly methylated genes (Seymour & Gaut, 2019; Takuno & Gaut, 2012). Also, since GbM genes are more prone to spontaneous mutations because of frequent deamination of C to T (Bird, 1980; Pfeifer, 2006), it is expected that such genes would evolve at a faster rate to consolidate for potential deleterious effects of such mutations. However, several studies have found that GbM genes evolve slowly (Takuno & Gaut, 2012, 2013; Takuno et al., 2017). Therefore, we investigated these specific features in pigeonpea and found that average length of GbM genes was 7.3 kb (Figure 2f), which was about two‐fold higher than the non‐methylated genes. The GbM genes in pigeonpea were found to be slightly higher in length than those of soybean (5.6 kb). To decipher the evolution rate among GbM genes in pigeonpea and soybean, Ks and Ka rates were calculated. We found that Ka (0.069, CG methylated; 0.12, non‐methylated) and Ks (0.30, CG methylated; 0.49, non‐methylated) were significantly low in GbM genes (*p* value < 2.2 × 10^−16^, Wilcoxon rank sum test) than the non‐methylated genes (Figure 2g,h).

#### 3.2.1 Gene‐body methylation exerts heterogeneous effects on gene expression

**FIGURE 3:**
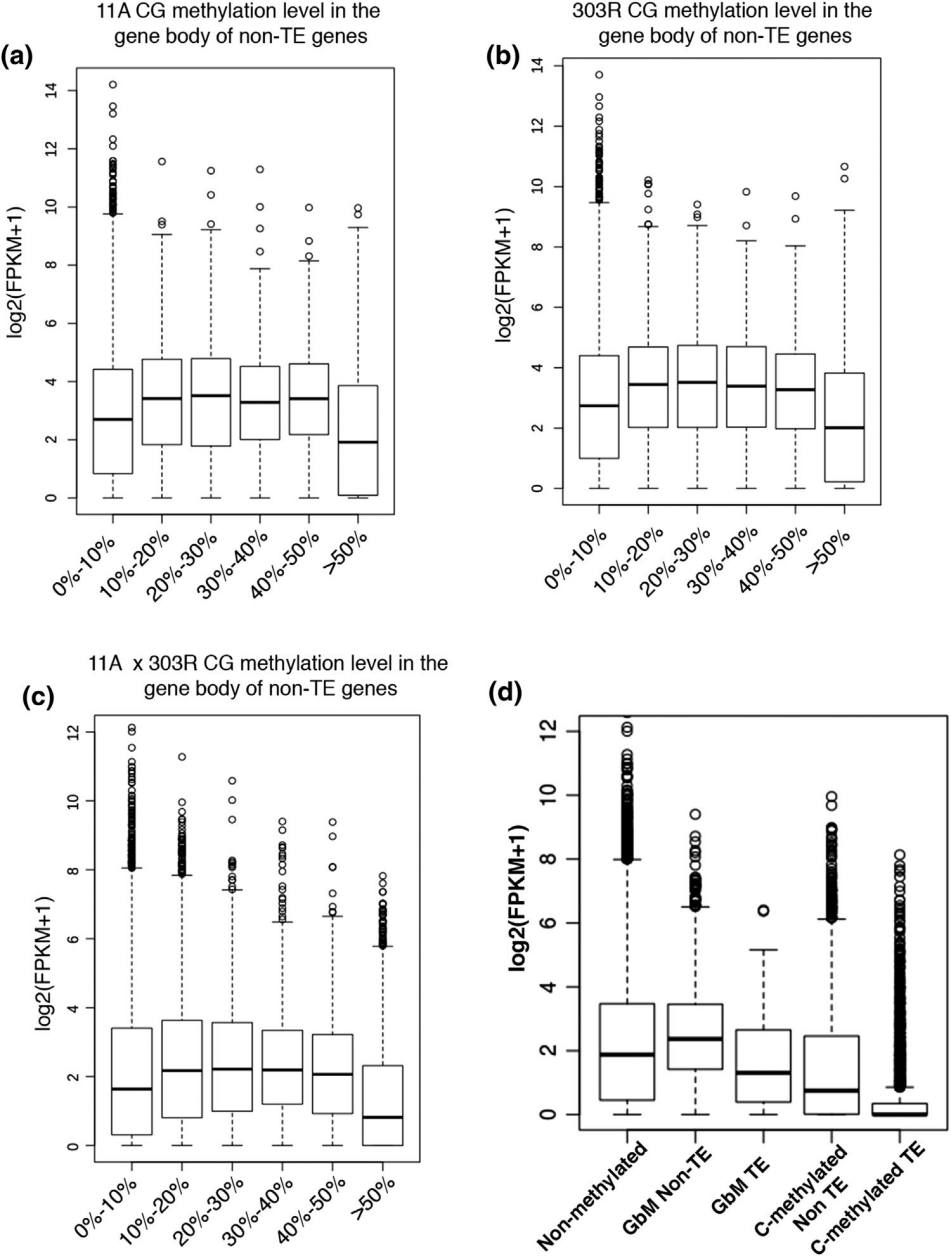
Correlation between CG gene methylation and expression levels. (a–c) Genes were classified based on CG methylation levels displayed along *x* axis and their normalized expression levels plotted on *y* axis in three genotypes of pigeonpea (a) 11A, (b) 303R, and (c) hybrid. Genes were divided into six groups. The sixth group contained genes with >50% methylation. Note a positive correlation of genes with CG methylation <0.4 and a negative correlation in mCG gene‐body methylation >0.5 between expression was found. (d) Boxplots showing normalized expression log2(FPKM+1) values of each gene category in pigeonpea. The significance difference in expression level was calculated using Wilcoxon rank sum test (*p* values details in main text)

A conclusive association between body methylation and gene expression remains obscure (Cokus et al., 2008; Hollister et al., 2011; Lister et al., 2008; Yang et al., 2011). In order to gain extensive insights into the association between GbM and global gene expression patterns, we categorized non‐TE genes based on their methylation levels and compared their expression patterns using RNA‐seq data of the three pigeonpea lines. We observed that moderately CG‐methylated genes (10–40%) tend to have highest expression level, whereas heavily methylated (>50%) showed lowest expression (Figure 3a–c). A spearman correlation coefficient was computed between body methylation and expression level to statistically test this observation. Genes with moderate levels of methylation showed a positive correlation, whereas genes with high methylation levels showed a strong negative correlation. The correlation varied from 0.09 to 0.18 (*p* value < 2.2 × 10^−16^) within moderately methylated genes and −0.43 to −0.48 (*p* value < 2.2 × 10^−16^) in heavily methylated genes. In summary, this result suggests that intermediate levels of GbM may have a positive impact on gene expression whereas high methylation likely represses transcription (Figure 3a–c). We also investigated the relationship between gene expression and transposons insertion in GbM and C‐methylated gene categories of pigeonpea (Figure 3d). We found that overall, GbM genes showed a higher expression than C‐methylated genes irrespective of transposons insertion in pigeonpea. Furthermore, C‐methylated genes with transposons inserted in their body region showed a significantly reduced expression among different gene categories (Wilcoxon rank sum test; *p* value < 2.2 × 10^−16^).

### 3.3 CG gene‐body methylation is conserved in homologous genes

**FIGURE 4:**
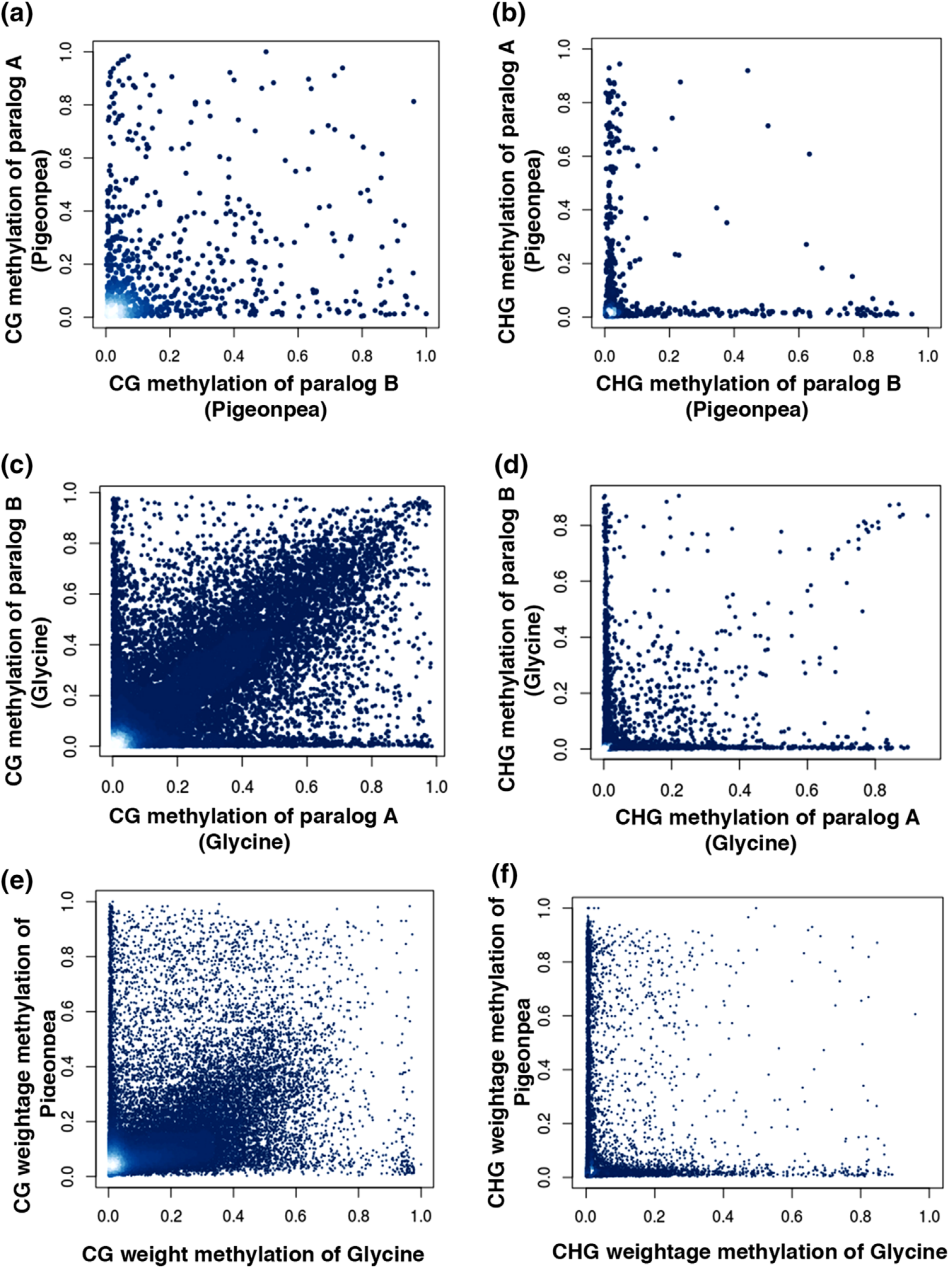
Correlation of DNA methylation level of homologous gene pairs in pigeonpea and soybean. (a–f) Pairwise comparison of CG and CHG weighted methylation levels in (a–d) paralogous gene pairs and (e–f) orthologous gene pairs. Spearman correlation coefficient was calculated to show association (details in main text)

Most legumes diverged from a common tetraploid ancestor ∼60 MYA that resulted in retention of thousands of duplicated genes. In order to understand the impact of DNA methylation after the events of speciation and soybean specific polyploidy, GbM within the orthologous and paralogous genes between pigeonpea and soybean was compared. We identified a total of 46,538 out of 95,711 genes (48.3%) as orthologous gene pairs between soybean and pigeonpea and 35,050 out of 54,324 (64%) and 4,636 out of 29,192 (16%) paralogous gene pairs in soybean and pigeonpea, respectively. For an explicit CG methylation comparative analysis, gene pairs containing C‐methylated genes (*P*_CHG_ < 0.05 or *P*_CHH_ < 0.05) were excluded based on earlier report (Kim et al., 2015). We found a high positive correlation in CG methylation levels of orthologous gene pairs (*r* = 0.53) as well as paralogous gene pairs of soybean (*r* = 0.69) and pigeonpea (*r* = 0.41) (Figure 4a,c,e). Notably, scatter plots revealed relatively high CG methylation levels in pigeonpea genes, consistent with the whole genome methylation levels. In contrast, we observed a relatively low positive correlation in non‐CG methylation levels between orthologs (*r* = 0.1 for CHG and *r* = 0.08 for CHH) and paralogs of pigeonpea (*r* = 0.19 for CHG and *r* = 0.1 for CHH) and soybean (*r* = 0.3 for CHG and *r* = 0.21) (Figure 4b,d,f; Supplemental Figure S4). A further meticulous analysis revealed that 4.3% of genes pairs (1,632 out of 37,109) in collinear blocks showed significant CG body methylation in both species, while only a small fraction, 0.9% of gene pairs, showed significant C‐methylation in both copies of genes. We found that a large proportion of orthologs, ∼69.9%, were non‐methylated in both species (20,826 of 29,757 in soybean and 11,742 of 16,781 in pigeonpea). These results reflect that during evolution, methylation state of non‐methylated genes likely remained robust. Next, to dissect methylation patterns at species level, we analyzed methylation status in synteny genes separately in soybean and pigeonpea. We found that ∼19.10% (5,685 of 29,757) of soybean and 8.42−10.31% of pigeonpea CG methylated genes (1,731 of 16,781 for F_1_ hybrid, 1,413 of 16781 for 11A, and 1,590 of 16781 for 303R) were present within the colinear orthologous regions. Altogether, our findings indicate that CG GbM patterns remained conserved between orthologs and paralogs of soybean and pigeonpea. Further, since our earlier analysis revealed that C‐methylated genes have high TE coverage (Figure 2d,e), our findings that non‐CG methylated genes are not highly conserved during evolution could possibly relate to a high frequency of TE insertion within C‐methylated orthologs. Additionally, the observed methylation patterns also indicate that, perhaps, evolutionary forces favored a relatively high conservation of CG body methylation in soybean than in pigeonpea during speciation.

We next sought to determine functional categories of GbM orthologous genes between pigeonpea and soybean using GO enrichment analysis. The most significant GO terms included functional categories associated with nitrogen compound biosynthetic processes, nitrogen compound transport, nitrogen compound metabolic processes, as well as gene categories belonging to carbohydrate derivatives transport (Supplemental Table S2). Given the fundamental role of nitrogen (N) in plant growth, reproduction, and protein content of seeds, especially in legumes, it is compelling that GbM orthologs of the two most important legume crops that diverged ∼30 MYA is enriched for gene categories belonging to nitrogen metabolism, transport, and biosynthesis.

#### 3.3.1 Analysis of DNA methylation and expression patterns among paralogous genes

Plant genomes are repeatedly restructured during evolution, resulting in genes to acquire different origins. We next aimed to trace the role of DNA methylation in genes that originated from different modes of duplication during evolution. Genes were divided into five categories based on their mode of duplication: singletons, WGD, tandem, proximal, and dispersed. We found only 16.35% of genes were WGDs, whereas single‐gene duplicates (SGDs)—dispersed, proximal, and tandem—were more abundant in pigeonpea genome (Table 1). The abundance CG body methylated and C ‐methylated singleton genes (11% CG and 9.8% mC) was slightly higher than the WGD genes (9.3% CG and 7.5% mC). We also observed high proportion of C‐methylated genes in SGD events (Table 1).

**TABLE 1:** Distribution of methylated genes in gene category with different mode of origin

Gene category	Total (29,574)	mCG genes (2,763) 9.3%	mC genes (7,187) 24.3%
Singleton	2,804	336 (11%)	271 (9.8%)
Dispersed	16,913	1,678 (9.9%)	4,053 (23%)
Proximal	1,413	83 (5.6%)	427 (29.7%)
Tandem	3,225	188 (5.7%)	720 (22.3%)
Whole‐genome duplicated	4,836	452 (9.3%)	367 (7.5%)

**FIGURE 5:**
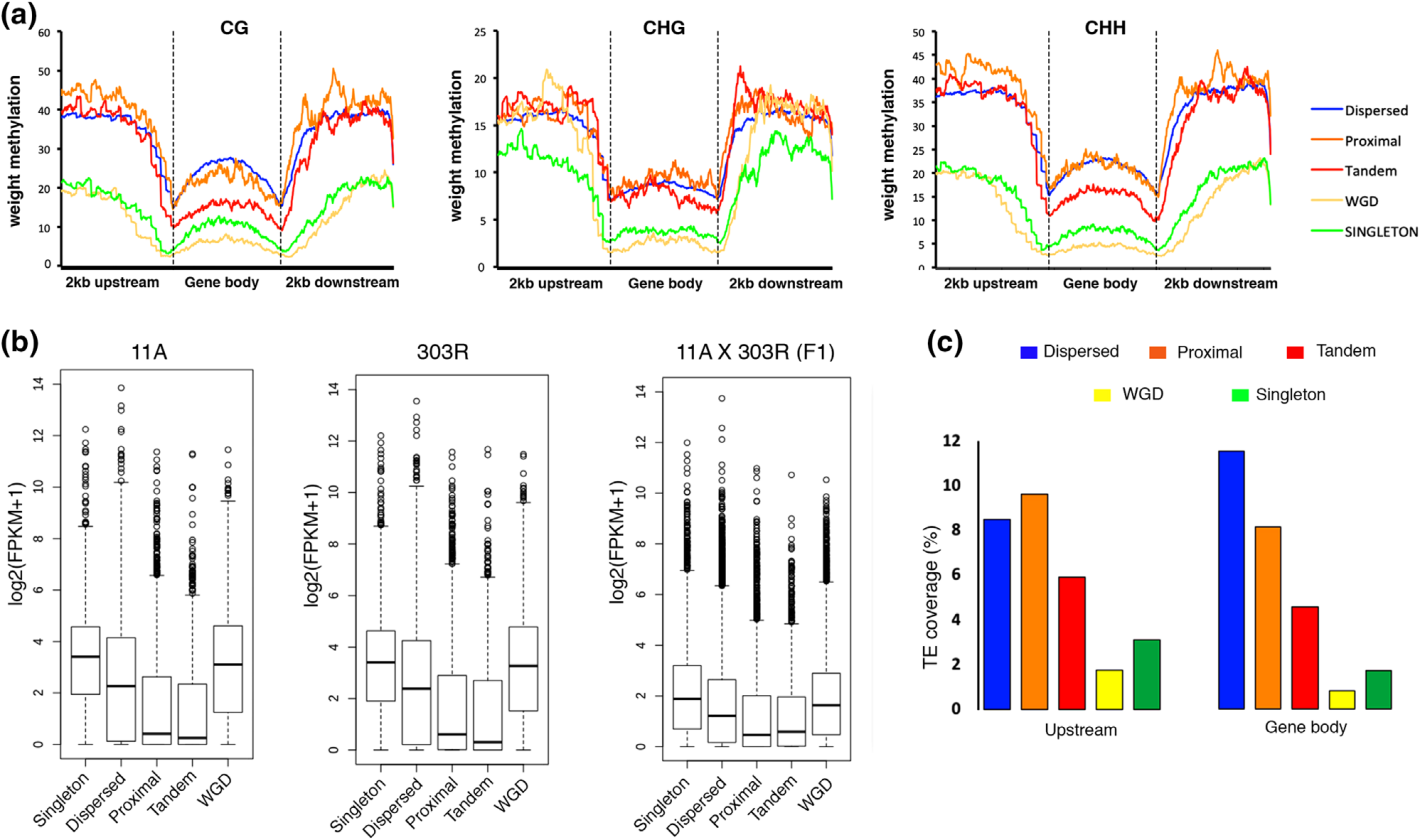
Gene‐body methylation and expression analysis in four different categories of whole‐genome duplicated (WGD) and single‐copy genes. (a) Line plots showing distribution of CG, CHG, and CHH weighted methylation in body and flanking region of different gene category. (b) Boxplots showing normalized expression levels (log2(FPKM+1) of genes in 11A, 303R, and hybrid lines. (c) Transposable element (TE) coverage (percentage) in gene‐body region of different genes categories

In order to further understand DNA methylation patterns in the genes derived from different duplication modes, we calculated and compared CG, CHG, and CHH weighted methylation levels of duplicated (SGD and WGD) and singleton genes. We found that genes belonging to dispersed group showed highest CG methylation level in the gene‐body regions (Figure 5a). The WGD group showed significantly low methylation levels relative to singleton genes (Wilcoxon rank sum test *p* value < 2.2 × 10^−16^). Within the duplicated gene categories, CG methylation levels within the body region of WGDs were significantly lower than other duplicated gene categories (dispersed, *p* value = 2.2 × 10^−16^; proximal, *p* value = 3.527 × 10^−5^; and tandem, *p* value = 6.836 × 10^−10^). We found that methylation patterns in CHG and CHH contexts were almost similar (Figure 5a). We also investigated if there were any resemblances or disparities in the expression levels of the genes based on their origin. We observed that expression level of singleton genes was significantly higher than duplicated genes (Wilcoxon test, *p* value < 4.083 × 10^−6^). Among duplicated groups, WGD genes showed significantly higher expression (Wilcoxon test, *p* value < 2.2 × 10^−16^) than the other duplicated genes (Figure 5b).

Genes with different duplication modes were found to be associated with distinct methylation and expression pattern. We next asked whether the methylation and expression patterns of duplicated genes were influenced by TE insertion. For this, TE coverage within gene‐body and upstream regions was calculated. We found a positive association between TE coverage and DNA methylation levels within five gene categories (Figure 5c). Consistent with the low CG and non‐CG methylation levels, WGD genes followed by singletons showed lowest TE coverage in both the upstream as well as gene‐body region. The dispersed and proximal genes showed maximal TE coverage in gene‐body regions, which correlated with highest CG and non‐CG methylation levels among all gene categories. Moreover, WGD and singleton showed negative association of gene expression and TE coverage. In contrast, no such association was observed among SGD groups.

### 3.4 Covariation of methylation and expression divergence in pigeonpea

**FIGURE 6:**
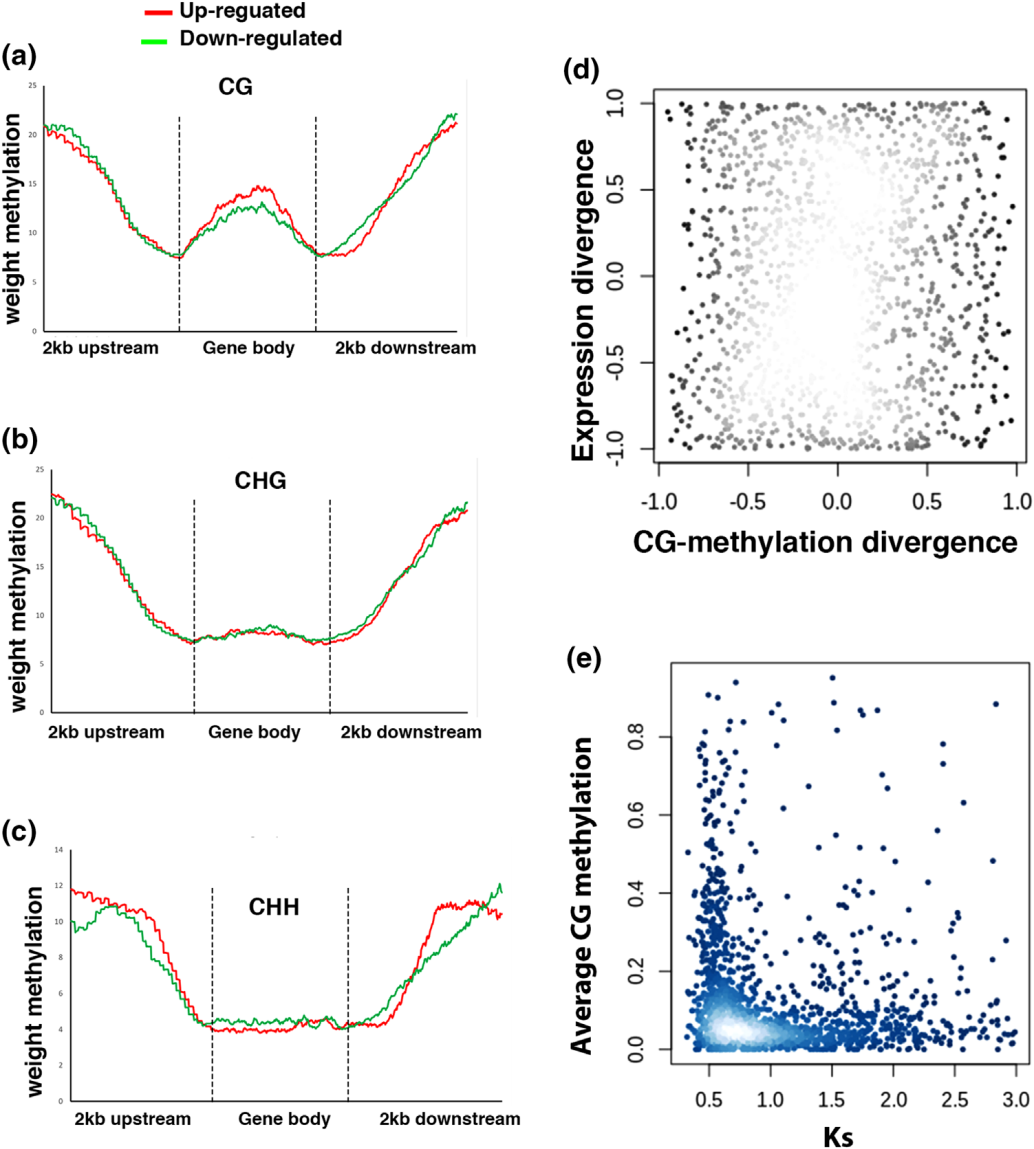
DNA methylation and expression divergence in whole‐genome duplication genes. (a–c) Metaplots showing methylation patterns in gene‐flanking (2 kb upstream and 2 kb downstream) and gene‐body regions of highly (log2FC > 2) and lowly (log2FC < −2) expressed duplicate gene pairs. Red and green lines represent highly and lowly expressed genes respectively. The significance test was performed using Wilcoxon rank‐sum test (*p* values in main text). (d) A scatter plot showing CG gene‐body methylation and expression divergence in duplicate genes. (e) A scatter plot showing relationship between CG gene‐body methylation and evolutionary age of paralog pairs with synonymous substitution rate (Ks) < 3

A previous study has reported a positive correlation between CG methylation and gene expression divergence in cassava (*Manihot esculenta* Crantz) (Wang et al., 2015). However, a nonsignificant relationship between CG methylation and expression divergence was found in tomato (*Solanum lycopersicum* L.) (Wang et al., 2018). Therefore, in order to further investigate this phenomenon in pigeonpea, we classified duplicated gene pairs as either highly or lowly expressed based on the fold change in the expression between gene pairs. We observed a positive association between CG body methylation and expression divergence, where highly expressed gene copies showed significantly (*p* value = 0.02, Wilcoxon rank sum test) high CG body methylation vs. lowly expressed genes (Figure 6a). No significant difference in CHG and CHH methylation was found across the body regions of differentially expressed genes in pigeonpea (Figure 6b,c).

Since our analysis above reveals a positive impact of CG body methylation on gene transcription in duplicated gene pairs, we further examined whether differential methylation levels could contribute to expression divergence among duplicate gene pairs. To test this, we calculated signed relative methylation (M1 − M2)/(M1 + M2) and expression divergence (E1 − E2)/(E1 + E2) in pigeonpea. We found a weak positive correlation (*r* = 0.07; *p* value = 0.006) of methylation and expression divergence, implying that the hypermethylated gene copy exhibited higher expression while hypomethylated copy tends to show lower expression (Figure 6d). Overall, this significance covariation of methylation and expression divergence suggests potential role of epigenetic difference in contributing to the retention of paralog, which may further contribute to substantiating their neofunctionalization during evolution. Moreover, we further examined whether DNA methylation influences evolution of duplicate gene pairs. To this end, average DNA methylation of paralogous genes were calculated, and then correlation analysis was performed between methylation level and Ks. We found a significant negative correlation (*r* = −0.25; *p* value < 2.2 × 10^−16^), implicating hypermethylation in younger duplicates (Figure 6e).

### 3.5 Natural epigenetic divergence among pigeonpea parental and F_1_ hybrid line

**FIGURE 7:**
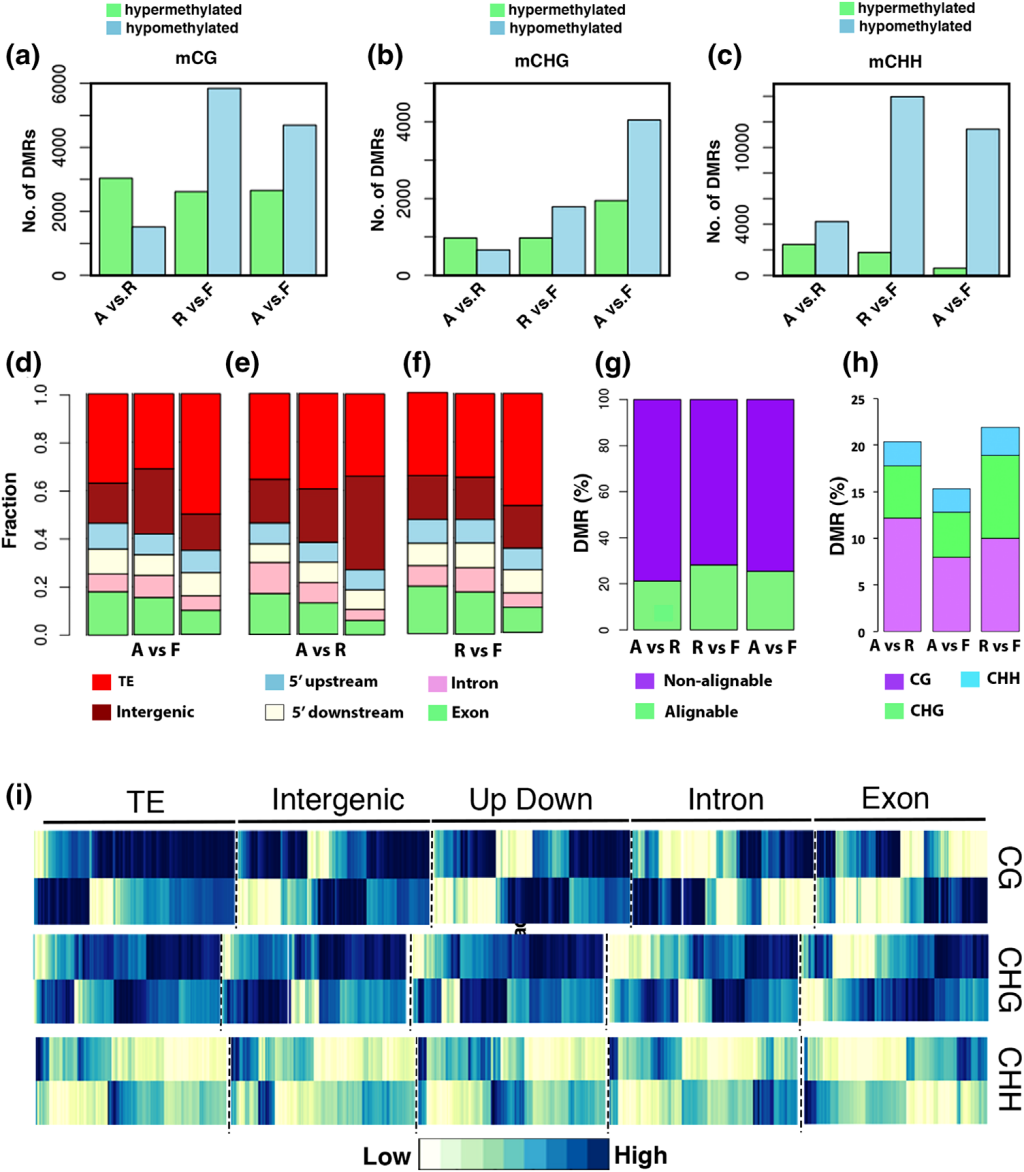
Characterization of DNA methylation variation among pigeonpea 11A, 303R, and hybrid lines. (a–c) Number of differentially methylated regions (DMRs) in (a) CG, (b) CHG, and (c) CHH contexts in 11A vs. 303R, 11A vs. hybrid, and 303R vs. hybrid. (d–f) Proportions of DMRs overlapping with annotated features of genome. (g) Stacked bar plot showing percentage of DMRs in aligned vs. nonaligned genomic regions of pigeonpea. (h) Stacked bar plot showing percentage of CG, CHG, and CHH DMRs in the aligned genomic regions of pigeonpea. (i) Hierarchical clustering (Pearson correlation method) of methylation level in 11A vs. 303R CG, CHG, and CHH DMRs in different genomic features

Naturally occurring epigenetic modifications, like DNA methylation, remains one of the fundamental sources of heritable variations within individuals of a species. By modulating gene expression, a stable inheritance of DNA methylation also underpins phenotypic variations often observed between genotypes (Becker et al., 2011; Raza et al., 2017; Schmitz et al., 2011; Zilberman et al., 2007). Therefore, we investigated the functional consequences of natural variation in DNA methylation in pigeonpea. We compared weighted methylation levels and calculated the number of DMRs in the three pigeonpea genotypes (Figure 7a–c). We found that, CHH DMRs were most enriched in all the three‐comparison scenarios (Figure 7c). Further classification of DMRs into hyper‐ and hypomethylated categories revealed a high abundance of hypermethylated DMRs in F_1_ hybrid line relative to parental lines in all the three cytosines contexts (Figure 7a–c). However, the whole genome CG‐ and CHG‐weighted methylation levels of the hybrid line were relatively low when compared with parental line.

We next examined the distribution of CG, CHG, and CHH DMRs within the different genomic elements. We found that most of the CG DMRs were localized in transposable element (35% in 11A vs. 303R; 37% in 11A vs. hybrid; and 34.5% in 303R vs. hybrid) followed by gene‐body region (18.4% in 11A vs. 303R; 18% in 11A vs. hybrid; and 19.7% in 303R vs. hybrid) and intergenic regions (17.8% in 11A vs. 303R; 16.6% in 11A vs. hybrid; and 18.1% in 303R vs. hybrid) (Figure 7d–f). A similar trend was observed for CHG and CHH DMRs distribution except in 11A vs 303R, where enrichment of CHH DMRs was found to be very frequent in intergenic regions vs. the rest of genomic regions. Furthermore, a subsequent hierarchical clustering of the DNA methylation in DMRs revealed that TE‐related DMRs displayed consistently high levels of CG and CHG methylation, whereas DMRs localized in the exon and intron regions showed relatively low levels of methylation (Figure 7i). Consistent with our predictions, DMRs in CHH context showed low methylation level among all clusters.

Lastly, we investigated if the DMRs could have possibly targeted the evolutionary conserved sequences. For this, the soybean and pigeonpea genomes were aligned in a 1:1 relationship and ∼90‐Mb sequences were found to be conserved. We found that a relatively low percentage of DMRs (20–28%) were located in the aligned portion of the genome (Figure 7g). We next investigated the conservation of CG, CHG, and CHH DMRs in the orthologs gene and again found a very low proportion (9.8% CG; 4.2% C‐DMR) of DMRs overlapping with the 986 and 711 orthologous genes, respectively (Figure 7h). These results indicate that perhaps the variably methylated marks in the genome, especially those that are C‐methylated, are not robustly maintained and are thus less likely to be conserved during evolution.

## 4 DISCUSSION

In this study, we performed an extensive comparative analysis of the floral bud methylome of two naturally varying pigeonpea lines: 11A (sterile), 303R (fertile), and their fertile F_1_ hybrid. To gain evolutionary insights, we also compared methylation patterns of pigeonpea with its close relative soybean that diverged 20‐30 MYA. Our analysis of the whole genome methylation patterns revealed highly stable methylation patterns at CG and CHG sites (symmetric sites) relative to CHH sites (asymmetric sites) of all the three lines of pigeonpea. We surprisingly found unexpected differences in patterns of CHH methylation in the gene and transposon density regions between the pigeonpea and soybean. Contrary to the observed general trends (Feng & Jacobsen, 2011) and to those that we observed in soybean, CHH methylation showed a negative correlation with transposon rich regions and a positive correlation with gene dense regions of pigeonpea genome. Occurrence of such dissimilarities in CHH methylation patterns have also been reported between *Brachypodium* and rice (Takuno & Gaut, 2013), two members of Poaceae family that diverged approximately 40‐53 MYA (The International Brachypodium Initiative, 2010). These findings collectively hint towards a possibility that molecular pathways regulating CHH methylation might not always be conserved between closely related species and may differ in some aspects.

Gene‐body methylation is an evolutionary conserved mechanism that was first discovered in crucifers (Tran et al., 2005). Although the functional roles of GbM have at large remained enigmatic, it has been proposed to be involved in regulating splicing and gene expression (Regulski et al., 2013; Tran et al., 2005; Zilberman et al., 2008). Our analysis in pigeonpea shows that genes with highest level of GbM exhibit lowest expression levels, and genes with moderate GbM levels show the highest expression. These observations seem to be in agreement with a previously proposed trade off where methylation within the gene body can curb the events of transcription initiation from cryptic promoter sites at the expense of transcriptional elongation (Zilberman et al., 2007). Gene body methylation genes are characterized by specific features such as higher gene length and slow evolutionary rates. We found that these features were maintained in both pigeonpea and soybean during evolution. However, our data show that pigeonpea GbM genes tend to be slightly longer than soybean GbM genes. Since we also found that soybean showed a relatively higher abundance of GbM genes than pigeonpea (Figure 2b), one possible causality of reduced gene length in soybean may be attributed to a recent WGD event independent of pigeonpea where evolutionary forces may have targeted GbM gene length to balance the effect of an additional WGD event on gene expression levels and to buffer any possible pleiotropic effects arising because of this event in soybean.

Evolutionary mechanisms, such as genome duplication followed by divergence, are fundamental to the evolution of genes in angiosperms. However, duplicated genes have been proposed to be constrained by the ‘loss and gain dilemma’ (Wagner, 1998, 2001), that is, they may diverge to acquire a new function or demote to pseudogenes and ultimately face gene loss. It has also been proposed that post duplication, reduction in the expression of genes mediated by epigenetic changes can contribute to a higher probability of their retention, thereby shifting the balance between loss and gain toward divergence and neofunctionalization (Chang & Liao, 2012; Rodin & Riggs, 2003). Since gene body methylation contributes to regulating expression levels, our results suggesting that gene‐body regions of young, duplicated genes were hypermethylated and those of older duplicated genes were hypomethylated (Figure 6e) are in line with the ‘expression divergence model’. These findings hint toward a potential role of the gene body methylation to help facilitate the retention of duplicated genes by expression regulation in addition to transcription regulation by promoter methylations (Keller & Yi, 2014).

A key question in plant breeding is to understand how the combined parental epigenome is regulated in hybrids to give rise to significantly different hybrid epigenome, which may alter genetic traits in hybrids. Therefore, it is important to understand the inheritance of parental epigenetic patterns in hybrids. We found that F_1_ hybrid line showed relatively low levels of the whole genome CG‐ and CHG‐weighted methylation compared with the parental lines. These observations are in line with a previously reported occurrence of nonadditive methylation events in plants (Greaves et al., 2012; Junaid et al., 2018) where methylation levels of hybrid at specific loci is different to parents (either increased or decreased). Further, since pigeonpea and soybean are closely related species, we investigated the methylation patterns of a diverse set of DMRs of pigeonpea (11A vs. 303R, 303R vs. F_1_ hybrid, and 11A vs. F_1_ hybrid) in soybean and found that DMRs are less likely to be targeted by the evolutionary conserved sequences.

Examination of functional GO categories in GbM orthologous genes showed a significant enrichment of genes involved in nitrogen compound synthesis, transport and metabolic processes, as well for carbohydrate derivatives transport. These results are compelling for two reasons. First, pigeonpea is an extremely important source of protein for human nutrition, and the above physiological processes are crucial in establishing protein content in seeds. Second, these findings hint towards a possibility where genes involved in nitrogen and carbohydrate metabolism were perhaps, repeatedly deployed or targeted for gene body methylation during evolution to help balance their expression for optimal function. In the future, it will be interesting to identify specific genes in these processes and generate epialleles by targeted epigenome editing using CRISPR technology (Gallego‐Bartolome et al., 2018) to generate pigeonpea cultivars with higher protein content without altering the DNA sequence.

## AUTHOR CONTRIBUTIONS

Alim Junaid: Conceptualization; Data curation; Formal analysis; Investigation; Methodology; Resources; Validation; Visualization; Writing – original draft; Writing – review & editing. Nagendra Kumar Singh: Supervision. kishor Gaikwad: Funding acquisition; Project administration.

## CONFLICT OF INTEREST

The authors declare no conflicts of interest.

## Supporting information

Supplemental Figure S1 Correlation and distribution of weightage methylation levels Scatter plot illustrating the correlation and distribution of weightage methylation levels in CG, CHG and CHH contexts between biological replicates of Pigeonpea.
Supplemental Figure S2. Comparative profile of DNA methylation variation across transposon regions in Pigeonpea and Soybean.
Supplemental Figure S3 Methylation pattern in T.E and Non‐TE gene categories.
Supplemental Figure S4 Comparative analysis of methylation between homologous gene pairs (A‐C) Comparison of CHH methylation level between paralog (A and B) and ortholog (C) pairs of Pigeonpea and Soybean.
Supplemental Table S1 Raw read summary of methylome and transcriptome data analyzed in this paper.

## Data Availability

The bisulfite and RNA‐seq reads generated in this study have been submitted in National Center for Biotechnology Information's Sequence Read Archive (SRA) database (http://www.ncbi.nlm.nih.gov/sra) under bio project PRJNA718971.
