# Supplementary material for: Evolutionary fates of gene‐body methylation and its divergent association with gene expression in pigeonpea
Source: Plant Genome. 2022 Jul 5;15(3):e20207. doi: 10.1002/tpg2.20207 (PMC12807044; doi:10.1002/tpg2.20207)
Supplement: Supplementary file 1 [file TPG2-15-e20207-s001.docx]

**Supplementary Figures and Tables.**

**Fig. S1 Correlation and distribution of weightage methylation levels** Scatter plot illustrating the correlation and distribution of weightage methylation levels in CG, CHG and CHH contexts between biological replicates of Pigeonpea. Methylation levels were calculated within 2kb windows across genome.

**Figure S2. Comparative profile of DNA methylation variation across transposon regions in Pigeonpea and Soybean**.

(A-L) Metaplots showing the distribution of mCG, mCHG and mCHH along the two different family of transposons, class I (DNA elements) and class II transposons (Copia, Gypsy and Lines) in Pigeonpea (red line) and Soybean (blue line). The flanking regulatory sequences (2kb upstream and 2 kb downstream) and gene body regions were divided into equal sized 20 bins and weightage methylation level were calculated for each bin.

**Fig S3 Methylation pattern in T.E and Non-TE gene categories.**

(A-D) Boxplots showing CG, CHG and CHH methylation level in non-TE and TE related genes in pigeonpea (A and B) and soybean (C and D).

**Fig S4 Comparative analysis of methylation between homologous gene pairs**

(**A-C**) Comparison of CHH methylation level between paralog (A and B) and ortholog (C) pairs of Pigeonpea and Soybean.

**Table S1 Raw read summary of methylome and transcriptome data analyzed in this paper.**

BS-Seq Reads and methylation Summary:

|  | Total Read pairs | Aligned Reads | Cytosine Coverage | Conversion efficiency | CG methylation (%) | CHG  methylation (%) | CHH methylation (%) |
| --- | --- | --- | --- | --- | --- | --- | --- |
| 11A-1 | 41444195 | 34499391 | 18.30X | 99.40 | 87 | 79.5 | 16 |
| 11A-2 | 44915161 | 35705077 | 19.80X | 99.60 | 88.51 | 79.74 | 14.4 |
| 303R-1 | 44919328 | 37335318 | 18.01X | 99.10 | 84 | 75.9 | 17.4 |
| 303R-2 | 48611751 | 40686140 | 23.96X | 98.20 | 84.2 | 75 | 16.8 |
| F1-1 | 78150290 | 57624389 | 20.34X | 99.10 | 78 | 63 | 15 |
| F1-2 | 71608011 | 51961701 | 14.51X | 99.40 | 80 | 68 | 17.5 |

RNA-Seq Reads Summary:

|  | Total Read pairs | Aligned Reads |
| --- | --- | --- |
| 303R1 | 42957061 | 35256940 |
| 303R2 | 29618927 | 24959794 |
| 11A1 | 68068793 | 55943835 |
| 11A2 | 41531572 | 29967453 |
| F1-1 | 20658784 | 17875261 |
| F1-2 | 17701384 | 12872010 |
